# Dual treatment approach for leiomyomas of urinary bladder: Experience from tertiary hospital

**DOI:** 10.1016/j.ijscr.2024.110316

**Published:** 2024-09-18

**Authors:** Geofrey Chiloleti, Isaack Mlatie, Salim Sobbo, Mariam A. Mbezi, Herry G. Kibona, Fransia A. Mushi

**Affiliations:** a Department of Surgery, School of Medicine, Muhimbili University of Health and Allied Sciences, Dar es Salaam, Tanzania; b Department of Urology, Muhimbili National Hospital, Dar es salaam, Tanzania; c Department of Pathology, Muhimbili National Hospital, Dar es Salaam, Tanzania

**Keywords:** Transurethral resection of bladder tumor, Cystoscopy, Leiomyoma, Bladder tumor, Ventral urethrotomy, Bladder neck

## Abstract

**Introduction and importance:**

Leiomyomas are rare, benign mesenchymal tumors. They represent 1 to 5 % of all urinary bladder tumors. 20 % are asymptomatic, but most presentations are voiding and storage, followed by hematuria. Surgery has been reported to be a standard treatment, depending on the size of the tumor and its location in the bladder. The following case report, we discuss the case of a bladder leiomyoma presenting with storage and voiding symptoms and managed with dual approach of Transurethral resection of bladder tumor (TURBT) and open urethrotomy.

**Case presentation:**

A 39-year-old female who presented with a one-year history of total hematuria and a blood clot that was ovoid in shape presented with urge incontinence, nocturia, strain during urination, and incomplete bladder emptying. She was mildly anemic, afebrile, with no palpable peripheral lymph node and no palpable mass. PVE revealed an anteriorly located cervix.

The patient's Labs revealed to have moderate anemia of 8 g/dl, blood chemistry was uneventful, and ultrasound (USS) revealed that both kidneys were normal. The urinary bladder was well distended, with a mass located at the base, measuring 4.13 cm × 4.14 cm. MRI revealed a well-circumscribed intramural tumor on the left side measuring 4.3 cm × 4.2 cm, close to the bladder neck.

A cystoscopy was used to visualize the tumor from the left lateral wall at 3 o'clock, extending to 5 o'clock, and part of the bladder neck. The tumor was solid, easily bled, and had an irregular margin, and the bladder mucosa was normal.

The 1st TURBT was used for diagnosis; although she still persistent storage and voiding symptoms, she subsequently underwent 2nd TURBT, which was resected to completion with the aid of ventral urethrotomy. After the TURBT, tumor protruded into the urethra, complete excision was performed through the urethra due to extension of the tumor to the urethra. The patient's postoperative events were uneventful, and the patient was discharged with catheter care for 10 days. On follow-up, hematuria resolved, and there were no lower urinary tract symptoms.

**Clinical discussion:**

Leiomyoma of the urinary bladder is a rare, benign mesenchymal tumor. They are the most common type of tumor of the urinary bladder. The most common presentations are storage and voiding symptoms, and hematuria. The initial USS can be used, and CT IVU or MRI is necessary for surveillance of the upper tract, possibly with respect to the tumor location in relation to the ureteric orifice. Tumors can be endovesical, intramural or extravesical, resulting in different presentations. Some tumors are pedunculated and can move close to the bladder neck or even at the urethra and present with urinary retention. Leiomyomas are surgically excised, and sometimes via a dual approach, transurethral resection of bladder tumors is important. These patients have a very low recurrence rate and are symptom free, and they have a good prognosis.

**Conclusion:**

Leiomyomas are surgically excised, and sometimes via a dual approach, transurethral resection of bladder tumors is important. These patients have a very low recurrence rate and are symptom free, and they have a good prognosis.

## Highlights

• Transurethral resection of bladder tumor
• Cystoscopy
• Leiomyoma
• Bladder tumor
• Vental urethrotomy

## 1 Introduction and importance

Leiomyomas are rare, benign mesenchymal tumors. They represent 1 to 5 % of all urinary bladder tumors [1]. 20 % are asymptomatic, but most presentations are storage and voiding symptoms, followed by hematuria [2]. Surgery has been reported to be a standard treatment, depending on the size of the tumor and its location in the bladder [3]. Some cases are in favorable locations and small, and transurethral resection of the bladder tumor can be performed [3].

## 2 Case presentation

We reviewed the case of 39-year-old, female. The patient presented to the urology clinic with complaints of blood in the urine for the past year, gradual onset, total hematuria associated with on and off blood clots amorphous in shape, no passage of tissue shreds, no exposure to radiation, chemical industries, cigarette smoking, and no history of postcoital bleeding.

On physical examination, the patient was pale, afebrile, and had no palpable peripheral lymph nodes; on the abdomen, the abdomen was obese, the hypogastrium was tender, and there was no palpable mass. Per vagina exam revealed an anteriorly located cervix.

**Fig. 1:**
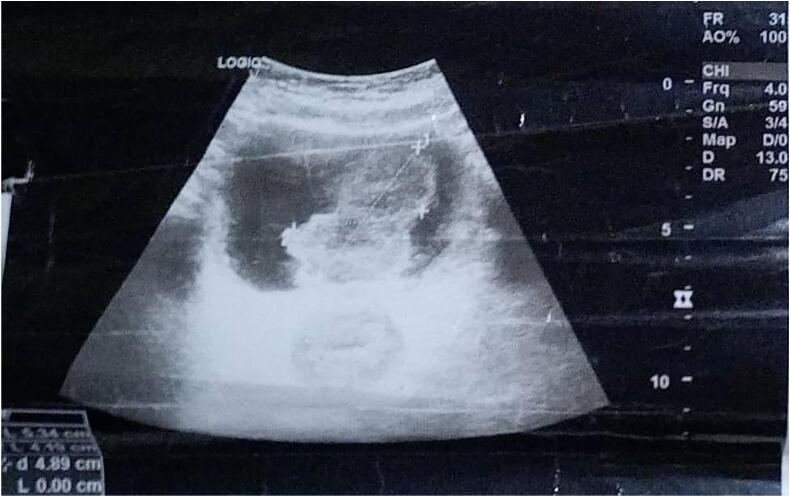
USS shows urinary bladder well distended, with a mass located at the base, measuring 4.13 cm × 4.14 cm.

Upon work-up, moderate anemia of 8 g/dl was revealed, and ultrasound revealed that both kidneys were normal with good corticomedullary differentiation and no calculi or hydronephrosis. The urinary bladder was well distended, with a mass located at the base, measuring 4.13 cm × 4.14 cm (Fig. 1).

**Fig. 2:**
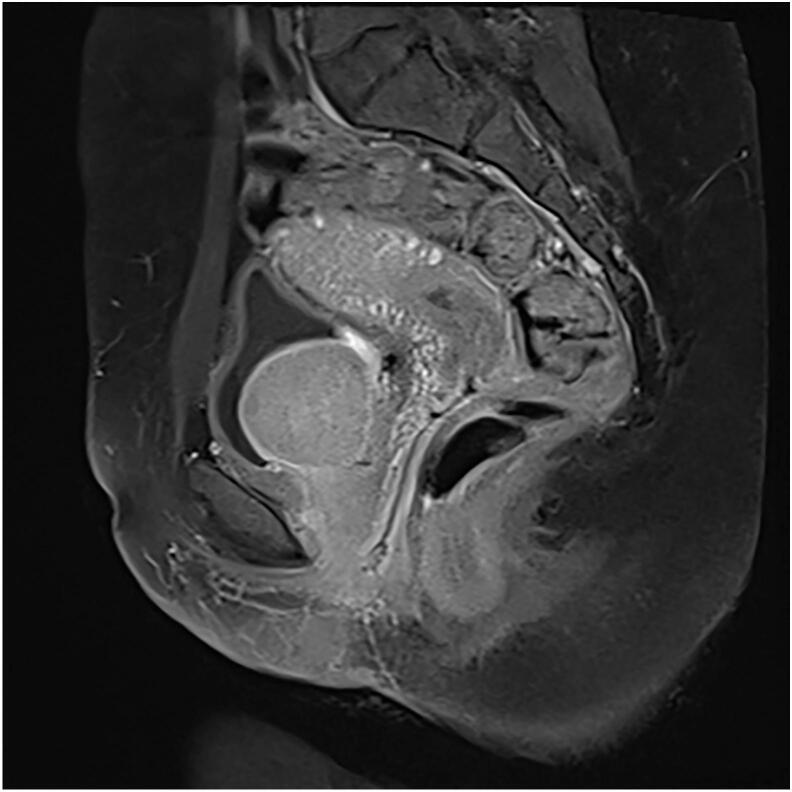
T2 weighted, MRI sagittal view, revealed a well-circumscribed intramural tumor on the left side measuring 4.3 cm × 4.2 cm.

MRI revealed a well-circumscribed intramural tumor on the left side measuring 4.3 cm × 4.2 cm, close to the bladder neck in Fig. 2. The patient was subjected to cystoscopy, which revealed a normal urethral meatus, a normal urethra, visualization of the tumor from the left lateral wall at 3 o'clock, extending to 5 o'clock, and part of the bladder neck. The tumor was solid, easily bled, and had an irregular margin. Partial resection of the bladder tumor was performed for diagnostic purposes, and tissue histology revealed tissue fragments lined with urothelium composed of stromal spindle cell proliferation in fascicles of elongated cells with eosinophilic cytoplasm and centrally located cigar-shaped nuclei with inconspicuous nucleoli. There was no cellular atypia or abnormal mitotic figures. These findings are consistent with the diagnosis of urinary bladder leiomyoma (Fig. 5, Fig. 6).

**Fig. 3:**
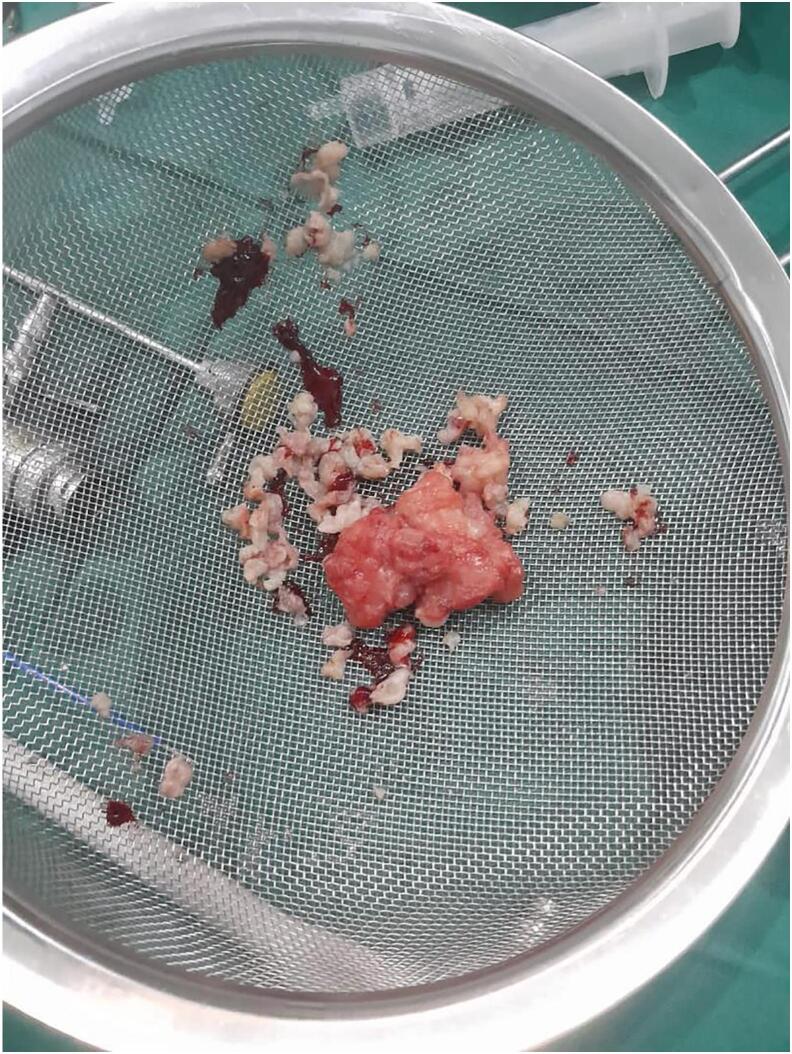
A specimen after TURBT.

**Fig. 4:**
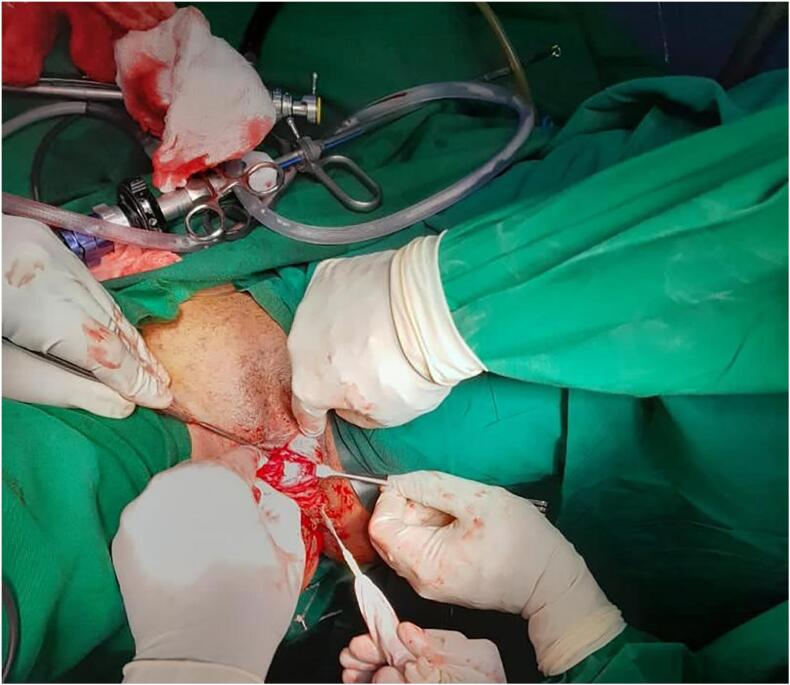
A surgical excision through ventral urethrotomy.

The bladder tumor was subsequently resected via transurethral resection, which was completed with the aid of ventral urethrotomy, due to extension of the tumor to the urethra for the second revisit cystoscopy (Fig. 3, Fig. 4).

**Fig. 5:**
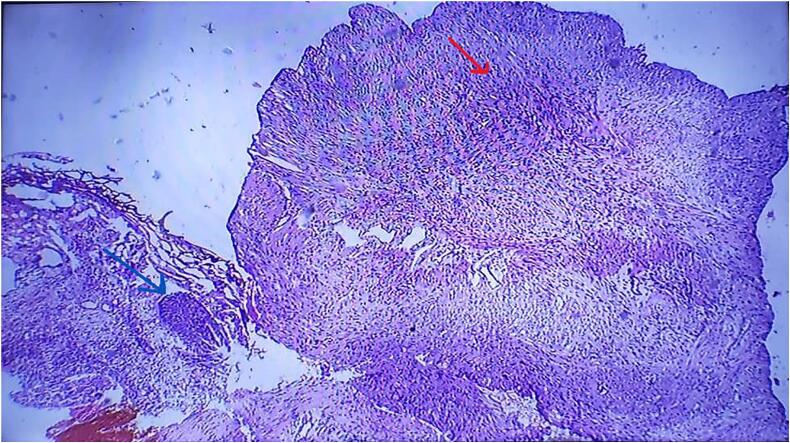
Spindle cellular tumor in fascicles (red arrow) and remnant of urothelial epithelium (blue arrow) ×10.

**Fig. 6:**
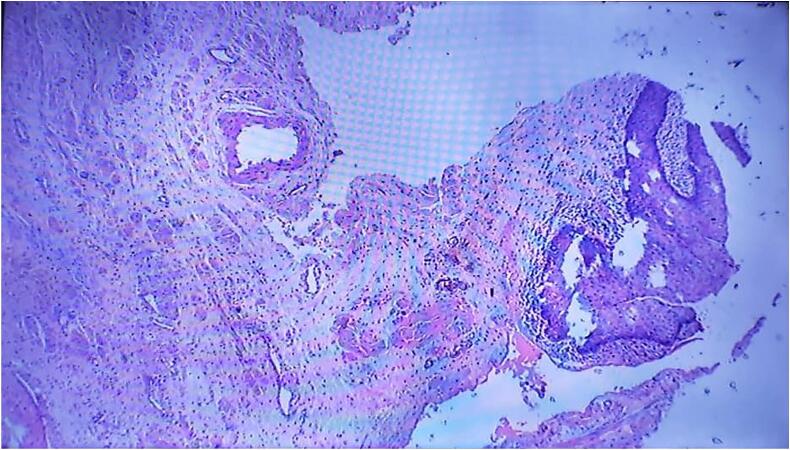
Slide shows spindle cells with eosinophilic cytoplasm, centrally located cigarshaped nuclei with inconspicuous nucleoli. No atypical mitotic figures seen. ×40.

The patient's postoperative events were uneventful, and the patient was discharged with catheter care for 10 days. On follow-up, hematuria resolved, and there were no lower urinary tract symptoms.

## 3 Clinical discussion

Leiomyoma of the urinary bladder is a rare, benign mesenchymal tumor. They are the most common type of tumor of the urinary bladder. The most common presentations are storage and voiding symptoms, and hematuria [1]. With respect to the use of a cystoscopy, other investigators reported that they tend to be larger, have smooth surfaces, and sometimes have difficulty passing to the bladder; additionally, other investigators reported that they are being excised trans-vaginal. For our patient, ventral urethrotomy was necessary to complete the excision of the tumor after resection of the bladder tumor. Therefore, both approaches are necessary for complete excision.

Ct scan ivu or MRI is necessary for the surveillance of the upper tract, possibly with respect to the tumor location in relation to the ureteric orifice. Obstruction of the ureteric orifice often results in hydronephrosis. The USS is initially used when a bladder tumor, its location, and its size are suspected. Tumors can be endovesical, intramural or extravesical, resulting in different presentations. Some tumors are pedunculated and can move close to the bladder neck or even at the urethra and present with urinary retention [2].

Regarding the management of bladder leiomyoma, surgical resection is the gold standard of treatment. However, the method of bladder leiomyoma resection differs depending on the location of the tumor and its size, either laparoscopic, open, or TURBT [4]. To our current knowledge, TURBT remains the mainstay of treatment for small, endovesical, and easily accessible tumors. In the case of an unfavorable position or a huge tumor, other surgical options are available such as segmental resection, laparoscopic partial cystectomy, or cystoprostatectomy [1,5].

The Dual approach according to our case, TURBT was first Endoscopy option to be done first, but due too big urinary bladder tumor couldn't be resected into completion by TUR. Another reason, since it had a stalk, and during resection it was falling into urethra, making it difficult to resect, that's when the open option of ventral urethrotomy was employed.

Internal sphincter was spared, the incision was Vental urethrotomy 2 to 3 cm from external urethral meatus, to facilitate complete tumor excision. The patient remained continent since the incision was far from bladder neck. Currently it 6-month post op, Continent, and follow up check cystoscopy reveal no Tumor recurrence, it is eventful.

To our case of leiomyoma, was surgically excised via a dual approach, and transurethral resection of bladder tumors is important. This results in a very low recurrence rate and is symptom free, and these patients have a good prognosis [6].

## 4 Conclusion

In conclusion, since our patient had a huge endovesical tumor near to the bladder neck, where couldn't be resected with TURBT into completion, adding to open urethrotomy made successful in resecting and dissection the tumor without recurrence. Follow up is important to ensure symptom resolution and no recurrence of bladder tumor. This case report demonstrates a rare case of bladder leiomyoma where dual approach of TURBT and Ventral Urethrotomy without partial cystectomy was employed without recurrence of the tumor. We highlight the importance of multi approach in providing patient-centered care. The treatment of a leiomyoma is dependent on its size and location in relation to the bladder wall. Since they are benign, surgery must be employed. The prognosis is invariably favorable, and symptom-free postoperative and recurrent disease is rare.

## Method

The work has been reported in line with the SCARE criteria [7].

## Consent

Written informed consent was obtained from the patient for publication of this case report and accompanying images. A copy of the written consent is available for review by the Editor-in-Chief of this journal on request.

## Ethical approval

The present study was exempted by the Ethics Committee of The Affiliated Hospital of Muhimbili national hospital, Tanzania, as this paper reports a case that emerged during normal surgical practice.

## Funding

No funding concerning this article.

## Guarantor

Dr Isaack M Mlatie
Dr Herry G Kibona
Dr Fransia Mushi
Dr Mariam Mbezi.

## Research registration number

Nil.

## CRediT authorship contribution statement

Dr Geofrey Chiloleti – Assistant Surgeon, Study concept & writing Manuscript
Dr Isaack Mlatie – Chief Surgeon, Correction manuscript
Dr Herry G Kibona Assistant surgeon, Correction manuscript
Dr Fransia A Mushi -Supervisor
Dr Mariam A Mbezi - Pathologist
Dr Salim Sobbo – Post op care and follow up care.

## Declaration of competing interest

The authors declare that they have no competing interests.
